# Potential use of liquid biopsy in diagnosing oral malignancies

**DOI:** 10.6026/973206300213465

**Published:** 2025-10-31

**Authors:** Timbadiya Vijaykumar Mansukhbhai, Madhvika Patidar, Manjiri Chakor, Dakshayani Vijay Patil, Pawan Rebello, Gatha Mohanty, Rashmi Laddha

**Affiliations:** 1 Department of Oral Pathology and Microbiology, Rishiraj College of Dental Science and Research Centre, Bhopal, Madhya Pradesh, India; 2 Department of Oral Pathology and Microbiology, Government College of Dentistry, Indore, Madhya Pradesh, India; 3 Department of Oral and Maxillofacial Surgery, RD Dental College & Research Center, Nagpur, Maharashtra, India; 4 Department of Oral & Maxillofacial Pathology, Hazaribag College of Dental Sciences and Hospital, Hazaribagh, Jharkhand, India; 5 Department of Oral & Maxillofacial Pathology & Oral Microbiology, Sardar Patel Postgraduate Institute of Dental and Medical Sciences, Lucknow, Uttar Pradesh, India; 6 Department of Periodontics and Oral Implantology, Institute of Dental Science, Siksha 'O' Anusandhan Deemed to be University, Bhubaneswar, Odisha, India; 7 Department of Periodontology, RR Kambe Dental College and Hospital, Akola, Maharashtra, India

**Keywords:** Liquid biopsy, oral malignancies, circulating tumor DNA, circulating tumor cells, exosomes, early cancer detection, non-invasive diagnostics, oral squamous cell carcinoma

## Abstract

Liquid biopsy is a minimally invasive method with strong potential for detecting and monitoring oral cancers. By analyzing ctDNA, CTCs, exosomes and other tumor-derived biomarkers, it enables early detection, prognosis and treatment monitoring. Unlike tissue biopsy, it allows repeated sampling to track tumor burden and genetic changes over time. In oral squamous cell carcinoma, studies show good sensitivity and specificity for detecting mutations, methylation and protein markers. However, standardization, biomarker validation and cost-effectiveness remain challenges for clinical use.

## Background:

Oral cancers, especially oral squamous cell carcinoma (OSCC), are a major global health issue because of their high prevalence, virulence and dismal survival rates when diagnosed at later stages. The surgical and adjuvant treatments, although improved, still rely on early detection as the key to better patient outcomes [1]. Traditional methods of diagnosis, such as visual inspection, tissue biopsy histopathological analysis and imaging, are useful but extremely limited. Tissue biopsy, the present gold standard, is invasive, frequently accompanied by patient discomfort and restricted in its potential to sample tumor heterogeneity or track disease development in real time. In addition, follow-up repeated tissue sampling is impracticable and potentially will allow delayed detection of recurrence or metastasis [2]. Liquid biopsy is an emerging non-invasive diagnostics and monitoring modality that examines tumor-derived material found in body fluids like blood, saliva, or plasma. It includes the detection and analysis of circulating tumor cells (CTCs), circulating tumor DNA (ctDNA), cell-free RNA, exosomes and tumor-associated proteins [3]. These biomarkers reveal molecular information on tumor biology, including mutational patterns, epigenetic patterns and protein expression profiles. Notably, liquid biopsy has the benefit of real-time monitoring so that clinicians can monitor response to treatment, identify minimal residual disease and recognize molecular alterations that accompany therapeutic resistance [4]. In the case of oral cancers, liquid biopsy is particularly promising since saliva, easily accessible and an indicator of local tumor activity can be combined with assays based on blood to increase diagnostic sensitivity [5]. Research has shown that certain genetic mutations (e.g., TP53, PIK3CA), altered DNA methylation patterns and protein biomarkers in liquid biopsy samples from OSCC patients can be reproducibly identified. Their incorporation into clinical management might allow for earlier diagnosis, improved prognostication and tailored therapeutic planning, which could enhance survival and quality of life [6]. Even though liquid biopsy holds promise, there are a number of challenges to routine clinical application in oral cancer. They are variability in collection and handling of the sample, disparity in analytical platforms, non-uniform biomarkers and requirements of large-scale validation studies [7]. Cost-effectiveness studies are also needed to ascertain whether these methods can be implemented in population-wide screening programs or not, especially in resource-poor areas where oral cancer burden is maximal [8]. Therefore, it is of interest to outline the prospects of liquid biopsy in the diagnosis of oral cancers, noting its diagnostic sensitivity, prognostic significance and application towards personalized treatment pathways, while considering the current challenges and possible future directions for clinical implementation.

## Materials and Methods:

This narrative review was conducted to compile and critically analyze existing literature on the potential of liquid biopsy in diagnosing oral malignancies, with a particular emphasis on oral squamous cell carcinoma. A comprehensive search was performed across PubMed, Scopus, Web of Science and Google Scholar for articles published between January 2000 and December 2024, using a combination of search terms including "liquid biopsy," "oral cancer," "oral squamous cell carcinoma," "circulating tumor DNA," "circulating tumor cells," "exosomes," "salivary biomarkers," and "cell-free DNA." Only full-text articles in English were considered and the search included original research, systematic reviews and meta-analyses that provided relevant human data. Studies were included if they evaluated liquid biopsy-derived biomarkers in blood, saliva, or other bodily fluids for diagnostic, prognostic, or predictive purposes in oral malignancies. Articles focusing exclusively on non-oral head and neck cancers without specific subgroup analysis, case reports, conference abstracts without peer-reviewed full texts and animal model studies were excluded. The screening process involved an initial review of titles and abstracts to identify potentially relevant studies, followed by full-text evaluation to confirm eligibility. Data from the selected studies were extracted to capture information on study design, sample size, type of biomarker assessed, analytical methods used, reported diagnostic accuracy, prognostic significance and clinical applicability. The extracted data were synthesized narratively, with attention to patterns in biomarker performance, emerging analytical techniques and potential integration into existing diagnostic pathways. Given the heterogeneity of methodologies and outcome measures, a meta-analysis was not attempted. Instead, emphasis was placed on summarizing the strength of the current evidence, identifying validated biomarker candidates and highlighting key limitations and future directions in the application of liquid biopsy for oral cancer detection and monitoring.

## Results:

A total of 72 eligible studies were included in this narrative review, comprising clinical investigations, biomarker validation studies and translational research focusing on the diagnostic role of liquid biopsy in oral malignancies. The evidence demonstrates that circulating tumor DNA, circulating tumor cells, exosomes and salivary biomarkers have considerable potential for early detection, disease monitoring and prognostic assessment in oral squamous cell carcinoma. Studies utilizing advanced analytical platforms such as digital droplet PCR and next-generation sequencing reported higher sensitivity and specificity for mutation detection compared to conventional PCR-based methods. Saliva emerged as a valuable, non-invasive sample medium for detecting tumor-specific mutations, methylation patterns and protein markers with promising diagnostic performance. Blood-based assays provided broader tumor genomic profiles, useful for capturing systemic disease features and identifying actionable mutations. Longitudinal monitoring studies highlighted the utility of liquid biopsy in detecting minimal residual disease and early recurrence before radiological evidence. Although substantial progress has been made in biomarker discovery and assay refinement, heterogeneity in study designs, small cohort sizes and lack of standardized protocols limit direct clinical translation. Emerging evidence supports the integration of liquid biopsy into multimodal diagnostic workflows to complement histopathology and imaging.

Table 1 (see PDF) presents the characteristics of included studies, showing that clinical observational designs were most common, saliva and plasma were the most frequent sample types and digital droplet PCR was the most used analytical method. Table 2 (see PDF) shows the distribution of biomarker categories evaluated in oral malignancy research, highlighting that ctDNA was the most frequently studied target, followed by CTCs, exosomes and salivary proteins. Table 3 (see PDF) demonstrates the diagnostic performance of ctDNA-based assays, with digital droplet PCR and next-generation sequencing achieving high sensitivity, specificity and AUC values in mutation detection. Table 4 (see PDF) shows the diagnostic and prognostic performance of CTC detection methods, indicating good sensitivity and specificity along with correlation to overall survival and recurrence risk. Table 5 (see PDF) presents the diagnostic value of salivary biomarkers, with IL-8, TP53 mutations and p16 methylation showing high sensitivity and specificity in oral cancer detection. Table 6 (see PDF) demonstrates the diagnostic potential of exosome-derived biomarkers, where upregulated miR-21, miR-31 and overexpressed EGFR protein were strongly associated with tumor presence. Table 7 (see PDF) shows the comparative performance of saliva and plasma in ctDNA detection, indicating similar concordance with tissue biopsy, with saliva showing slightly higher detection rates. Table 8 (see PDF) presents the role of liquid biopsy in recurrence monitoring, showing that ctDNA, CTCs and exosomal miRNAs allowed earlier detection of recurrence compared to imaging modalities. Table 9 (see PDF) shows the frequency of use and advantages of analytical platforms, with ddPCR offering highest sensitivity, NGS enabling comprehensive profiling and qPCR providing a cost-effective alternative. Table 10 (see PDF) demonstrates the most cited barriers to clinical translation, including lack of standardization, limited biomarker validation, small cohort sizes and high cost/resource requirements.

## Discussion:

This review consolidates current evidence on the potential of liquid biopsy in diagnosing oral malignancies, with particular emphasis on oral squamous cell carcinoma. Across diverse study designs, sample sources and analytical platforms, the findings collectively highlight the diagnostic, prognostic and disease-monitoring capabilities of circulating tumor DNA, circulating tumor cells, exosomes and salivary biomarkers [9]. These minimally invasive tools offer unique advantages over conventional tissue biopsy, particularly in enabling repeated sampling, capturing tumor heterogeneity and providing real-time molecular insights. The predominance of observational and case-control studies illustrates the early-stage nature of this field, yet consistent trends emerge [10]. Circulating tumor DNA analysis, especially when performed using digital droplet PCR or next-generation sequencing, demonstrates high sensitivity and specificity in detecting common genetic alterations such as TP53 mutations and DNA methylation changes [11]. These platforms enable detection of tumor-specific alterations even at low abundance, making them valuable for early diagnosis and minimal residual disease detection. Saliva-based assays are of particular relevance in oral cancers due to their direct contact with tumor sites, offering a simple, non-invasive medium for identifying genetic and protein biomarkers with diagnostic accuracy comparable to blood-derived samples [12]. Circulating tumor cells add a complementary layer of information by allowing phenotypic and genotypic characterization of intact tumor cells shed into the bloodstream. Studies consistently link higher CTC counts with worse overall survival and increased recurrence risk, reinforcing their potential role as a prognostic biomarker. Microfluidic platforms and immunomagnetic enrichment methods have enhanced the sensitivity of CTC detection, though challenges remain in distinguishing malignant from benign epithelial cells and standardizing assay thresholds [13]. Exosome-derived biomarkers, including specific microRNAs such as miR-21 and miR-31 and proteins like EGFR, are increasingly recognized as promising indicators for early-stage disease detection. Their stability in biological fluids and role in tumor communication pathways make them attractive for clinical translation. However, heterogeneity in isolation techniques and the lack of consensus on reference markers hinder reproducibility across studies [14]. Comparative analyses between saliva and plasma for ctDNA detection suggest that both mediums provide high concordance with tissue biopsy results, with saliva often yielding higher detection rates for locally confined lesions. This highlights the value of dual-sample approaches that leverage both local and systemic biomarker sources, potentially improving overall diagnostic sensitivity. Furthermore, longitudinal monitoring studies reveal that liquid biopsy can detect recurrence weeks to months before radiological confirmation, creating a window for earlier therapeutic intervention [15].

The technological backbone of these advances relies heavily on high-sensitivity analytical platforms. Digital droplet PCR offers unmatched sensitivity for targeted mutation detection, while next-generation sequencing enables comprehensive genomic profiling, essential for precision oncology approaches. Real-world integration will likely require balancing these capabilities against cost, turnaround time and infrastructure availability [16, 17]. Despite the encouraging data, several barriers impede the clinical translation of liquid biopsy in oral malignancies. A lack of standardized protocols for sample collection, processing and biomarker quantification results in significant inter-study variability. Many studies are limited by small sample sizes, which constrain statistical power and limit generalizability [18]. The majority of validated biomarkers still require large-scale prospective validation across diverse patient populations. Cost and accessibility remain critical considerations, particularly in low-resource settings where oral cancer burden is disproportionately high. Addressing these challenges will require coordinated multi-institutional collaborations, investment in assay standardization and integration of cost-effectiveness analyses into future research designs [19, 20]. From a clinical standpoint, liquid biopsy holds the potential to complement rather than replace traditional diagnostic modalities. Its greatest value may lie in integration into multimodal diagnostic workflows, where histopathology, imaging and molecular analysis provide a comprehensive assessment of tumor biology and treatment response. In high-risk populations, liquid biopsy could be deployed as a screening tool to detect premalignant changes or early-stage lesions, particularly when combined with risk stratification algorithms [21]. Future research should focus on longitudinal, large-cohort studies to validate biomarker performance and assess their impact on clinical decision-making. Advances in assay automation, multiplexed biomarker detection and artificial intelligence driven data interpretation are likely to accelerate the pace of translation from research to practice. Cross-disciplinary partnerships between oncologists, pathologists, molecular biologists and bioengineers will be essential in overcoming current technical and logistical hurdles. In summary, liquid biopsy represents a transformative approach in the diagnosis and management of oral malignancies, offering minimally invasive, repeatable and molecularly informative alternatives to conventional methods. While significant progress has been made in biomarker discovery and validation, on-going efforts toward standardization, large-scale validation and cost optimization are critical to realizing its full clinical potential.

## Conclusion:

Liquid biopsy offers a promising, minimally invasive approach for the early detection, prognosis and monitoring of oral malignancies, with the potential to complement existing diagnostic and surveillance strategies. Continued efforts in biomarker validation, protocol standardization and cost optimization are essential to enable its effective integration into routine clinical practice and to improve outcomes for patients with oral cancers.

## References

[1] Naito Y, Honda K. (2023). J Pers Med..

[2] Lousada-Fernandez F (2018). Int J Mol Sci..

[3] Zhang X, Li B. (2023). Oral Dis..

[4] Baby NT (2022). Int J Oral Maxillofac Surg..

[5] Mazumder S (2019). Cancer Epidemiol..

[6] Palaia G (2022). J Clin Exp Dent..

[7] Gattuso G (2022). Noncoding RNA..

[8] Roi A (2023). Biomedicines..

[9] Rapado-González O (2019). Oral Oncol..

[10] Kinane DF (2024). Periodontol 2000..

[11] Smith RA (2020). Methods Mol Biol..

[12] Lam AK (2020). Methods Mol Biol..

[13] Goodson ML (2017). J Oral Pathol Med..

[14] Araújo ALD (2023). Curr Oncol Rep..

[15] Ishikawa R (2022). Curr Probl Cancer..

[16] Kujan O (2018). J Oral Sci..

[17] Alsarraf A (2018). J Oral Pathol Med..

[18] Liu J (2021). Biomed Res Int..

[19] Payne K (2024). Oral Oncol..

[20] Deuerling L (2019). Cancers (Basel)..

[21] Mahmood N (2021). Asian Pac J Cancer Prev..

